# Nucleophilic Synthesis of 6-l-[^18^F]FDOPA. Is Copper-Mediated Radiofluorination the Answer?

**DOI:** 10.3390/molecules25194365

**Published:** 2020-09-23

**Authors:** Raisa N. Krasikova

**Affiliations:** N.P. Bechtereva Institute of the Human Brain Russian Academy of Science, 197376 St. Petersburg, Russia; raisa@ihb.spb.ru

**Keywords:** ^18^F, 6-l-[^18^F]FDOPA, nucleophilic radiolabeling, copper-mediated, organoborons, organostannanes, iodonium salts, positron emission tomography

## Abstract

Positron emission tomography employing 6-l-[^18^F]fluoro-3,4-dihydroxyphenylalanine (6-l-[^18^F]FDOPA) is currently a highly relevant clinical tool for detection of gliomas, neuroendocrine tumors and evaluation of Parkinson’s disease progression. Yet, the deficiencies of electrophilic synthesis of 6-l-[^18^F]FDOPA hold back its wider use. To fulfill growing clinical demands for this radiotracer, novel synthetic strategies via direct nucleophilic ^18^F-radiloabeling starting from multi-Curie amounts of [^18^F]fluoride, have been recently introduced. In particular, Cu-mediated radiofluorination of arylpinacol boronates and arylstannanes show significant promise for introduction into clinical practice. In this short review these current developments will be discussed with a focus on their applicability to automation.

## 1. Introduction

Positron Emission Tomography (PET), based on the use of tracers labeled with short-lived positron-emitting radionuclides, is a well-established methodology for non-invasive molecular imaging of living subjects, used in both pre-clinical and clinical settings, particularly in the fields of oncology and neurology. Currently, PET imaging is an indispensable tool in the development of new drugs and is widely employed by both pharmaceutical companies and academic groups. Fluorine-18, a short-lived isotope of fluorine (T_1/2_ = 109.7 min) is the predominant PET radionuclide currently in use, as its relatively long half-life allows for complex multi-step syntheses providing access to a large variety of ^18^F-labeled radiopharmaceuticals. Following the enormous commercial success of the off-site distribution model for the 2-[^18^F]fluoro-2-deoxy-d-glucose ([^18^F]FDG) for diagnostic applications, a number of ^18^F-based radiotracers become available from centralized cyclotron-equipped production facilities for distribution to distant PET imaging units in different hospitals. Fast-growing clinical demand for ^18^F-labeled molecular imaging probes, especially in the central nervous system and oncology fields, provide a powerful stimulus for the development of new efficient radiolabeling strategies suitable for automated procedures that are mandatory in large-scale productions.

^18^F-fluorinated aromatic amino acids that were introduced as PET radiotracers in the early 1980s have been successfully used for decades in a great variety of pre-clinical and clinical studies [1,2,3]. Among them, the 6-l-[^18^F]fluoro-3,4-dihydroxyphenylalanine (6-l-[^18^F]FDOPA) has earned a distinguished position as one of the most recognized tracers due to a wide and expanding spectrum of application in clinical studies [4,5]. Since its introduction in 1983 [6] for the in vivo assessment the central dopaminergic function of presynaptic neurons, 6-l-[^18^F]FDOPA is regarded as the “gold standard” for the detection and post-treatment monitoring of Parkinson’s disease (PD) [7,8] as well as other neurodegenerative disorders [9,10]. Following metabolic pathway of L-DOPA, the ^18^F-fluorinated analogue crosses the blood–brain barrier by facilitated diffusion and is subsequently decarboxylated by the aromatic-l-amino acid decarboxylase (AADC) to 6-[^18^F]fluorodopamine, which is retained within vesicles of dopaminergic neurons. About a decade ago [11], applications for this radiotracer for use in oncologic imaging have been demonstrated, following the introduction of the fused PET/CT methodology. PET/CT scanning with the 6-l-[^18^F]FDOPA has been shown to be a valuable technique for imaging a variety of neuroendocrine tumors (NETs) such as pheochromocytomas and paragangliomas, medullary thyroid carcinoma and for resolving causes of primary hyperinsulinemia in pediatric patients and adults [4,12,13,14]. In addition, this radiotracer has been shown to produce accurate results in detection of primary and recurrent high- and low-grade cerebral gliomas [15,16,17,18,19]. PET imaging with 6-l-[^18^F]FDOPA and two other amino acid tracers—l-[^11^C-methyl]methionine and *O*-(2-[^18^F]fluoroethyl)-l-tyrosine—was recommended by the Response Assessment in Neuro-Oncology (RANO) working group as a complementary tool for assessing gliomas at initial diagnosis and following management [18].

**Scheme 1:**
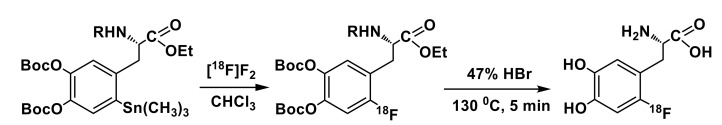
Typical electrophilic synthesis of 6-l-[^18^F]FDOPA via fluorodestannylation reaction (R = Boc or CHO).

As a result, the number of requests for clinical 6-l-[^18^F]FDOPA PET and PET/CT studies has been increasing dramatically in the recent years, despite of relatively high cost of single radiotracer dose and costs of studies themselves. The use of this radiotracer is held back, to a great extent, by the absence of a simple and efficient production method, that could, similarly to the [^18^F]FDG, provide Curie-level amounts of tracer using fully automated nucleophilic radiofluorination reactions, utilizing [^18^F]fluoride that is easily available from water cyclotron targets. However, until relatively recently, production methods for 6-l-[^18^F]FDOPA as well as other ring-fluorinated ^18^F-labeled amino acids relied primarily on the electrophilic route [20] employing [^18^F]F_2_ available from ^20^Ne(d,α)^18^F or ^18^O(p,n)^18^F nuclear reactions conducted in cyclotron gas targets. Presently, the synthesis of 6-l-[^18^F]FDOPA for clinical applications is conducted chiefly via an electrophilic regioselective ^18^F-fluorodemetallation [21] of commercially-available organotin precursors using gaseous [^18^F]F_2_ (Scheme 1). Despite the apparent simplicity and adaptability to modern automated synthetic modules [22,23,24] the electrophilic approach is known to have several limitations, such as difficulty in handling of radionuclide in a gas form (as [^18^F]F_2_), low productivity of the cyclotron targets and unavoidable addition of [^19^F]F_2_ carrier gas leading to decrease of molar activity (A_m_) of the radiotracers produced [25]. Even though the molar radioactivity does not appear to be of a critical importance for clinical application of 6-l-[^18^F]FDOPA, it is still desirable for obtaining high quality PET images [26] and ensuring absence of adverse reactions in patients [27]. One approach to improve the productivity and A_m_ of electrophilic method has been a post-target generation approach based on the conversion of nucleophilic [^18^F]fluoride into [^18^F]F_2_ gas in an electrical discharge chamber, introduced by Solin’s group [28,29]. This advanced methodology allowed production of radiotracers with A_m_ exceeding 15 GBq/μmol at the end of synthesis and in clinically useful amounts [30]. In a more recent study by the Solin and Gouveneur groups, the [^18^F]F_2_ obtained with high A_m_ was used to prepare [^18^F]selectfluor bis(triflate) [31], which was followed by its application in the electrophilc synthesis of 6-l-[^18^F]FDOPA with aryl boronic ester as a labeling precursor. However, the method described was complicated and its application limited to Turku PET center facilities, as it required rather specific equipment and non-trivial operation of an electric discharge cell [28,29], or, as in the more recent iteration, the use of an UV laser [30]. For these technical reasons, the wider adoption of this method proved to be difficult and it remains a rather esoteric example of possible synthetic approaches to production of 6-l-[^18^F]FDOPA. To date, most of the preparation of clinically useful doses of 6-l-[^18^F]FDOPA are performed in electrophilic reactions employing “conventional” [^18^F]F_2_ (Table 1). The quality requirements are listed in the EP monograph (Fluorodopa, ^18^F, prepared by electrophilic substitution) [32]: radiochemical purity ≥ 95%; enantiomeric purity ≥ 98%; 6-l-[^19^F]FDOPA (“cold” FDOPA)—not more than 15 mg/V; DOPA—not more than 1 mg/V; Me_3_SnCl < 50 ppm; pH 4.0 -5.5. Although substantial improvements in the 6-l-[^18^F]FDOPA production capability was achieved using the proton irradiation of enriched oxygen gas (^18^O(p,n)^18^F) instead of ^20^Ne(d,α)^18^F) nuclear reaction [24], the starting activities that can be obtained for [^18^F]F_2_ methods are still far below than those of [^18^F]fluoride generated from proton bombardment of ^18^O-enriched water targets.

Recent developments in radiofluorination methodologies including traditional multi-step nucleophilic aromatic substitution (S_N_Ar) reactions and novel “late-stage” transition metal-mediated approaches offer new routes for the introduction of fluorine-18 into non-activated arenes (and thus to the labeling of ring-fluorinated aromatic amino acids). Although advancements made in the preparation of 6-l-[^18^F]FDOPA have been the subject of several reviews published between 2014 and 2017 [33,34,35,36], this field has progressed tremendously since. It can be illustrated by an excellent review paper by Scott and co-workers, demonstrating the advances achieved within last 5 years in the field of labeling various arenes via Cu-mediated radiofluorinations [37].

This short review will focus exclusively on the preparation of a single but very important radiotracer—6-l-[^18^F]FDOPA—through the nucleophilic route employing various late-stage transition metal-mediated approaches with a specific attention to the recent developments in the copper-mediated fluorination methods and the aspects of their automation which is an unavoidable necessity when it comes to wider clinical application.

## 2. General Concepts for Nucleophilic Synthesis of 6-l-[^18^F]FDOPA

**Scheme 2:**
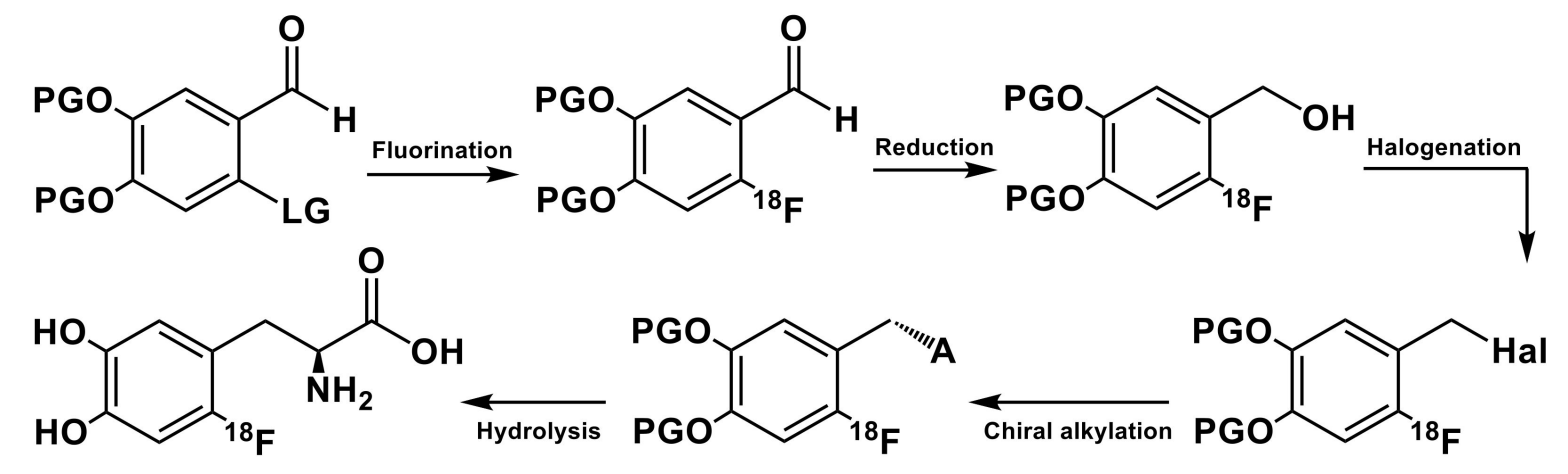
General route to asymmetric synthesis of 6-l-[^18^F]FDOPA: PG (protecting groups)—methoxy, methylenedioxy; LG (leaving group)—NO_2_, N(CH_3_)_3_^+^Tf^−^; Hal = Br, I; A—chiral auxiliary.

Given the numerous drawbacks associated with the production and use of [^18^F]F_2_ [26], the majority of ^18^F-fluorinations currently in use are developed following nucleophilic routes [38,39,40,41]. For this purpose, the [^18^F]fluoride is widely available as a starting material in amounts of up to 300 GBq through ^18^O(p,n)^18^F nuclear reaction in high-pressure ^18^O-enriched water targets [42]. The obtained no carrier added (n.c.a.) [^18^F]fluoride is trapped on a strong anion-exchange resin and then eluted, typically with acetonitrile/water solution of a phase-transfer catalyst (PTC) and base; reactive [^18^F]fluoride species are then obtained by azeotropic drying to facilitate removal of the residual water. However, while nucleophilic production methods generally allow production of larger quantities of radiotracers with lower costs, their application to the synthesis of 6-l-[^18^F]FDOPA and other aromatic amino acids was not as straightforward as labeling of the [^18^F]FDG and other aliphatic substrates. Traditional nucleophilic aromatic substitution (S_N_Ar) reactions, including those using [^18^F]fluoride as the nucleophile, require arene substrates with strong electron-withdrawing groups in the *ortho*- or *para*-positions of the aromatic structure combined with an appropriate leaving group. Therefore attempts to introduce [^18^F]F^−^ into aromatic nuclei of amino acids via direct nucleophilic route generally fails as the phenyl ring lacks electron-withdrawing groups. Therefore, the labeling must be approached through multi-steps “built-up” procedures and asymmetric synthetic strategies based on the alkylation of activated C-H acid glycine fragments incorporated in bulky structures possessing steric hindrance relating to one of two possible directions of the nucleophilic attack. Such asymmetric approaches provided access to 6-l-[^18^F]FDOPA in high enantiomeric purity which is a prerequisite for PET applications. Fluorine-18 is introduced into the aromatic ring of the substituted benzaldehyde containing an appropriate leaving group, followed by reduction and halogenation reactions. ^18^F-labeled benzyl halide thus obtained was then used in the key chiral alkylation step (Scheme 2) followed by deprotection with 57% HI at 180 °C.

One of the first stoichiometric asymmetric synthetic approaches via diastereoselective alkylation of chiral glycine enolates was suggested by Lemaire and co-workers as early as 1994 [43]. Practically, this method was not suitable for automation because the key alkylation step had to be performed under extreme reaction conditions, employing very strong bases (LDA), low temperatures and strict anhydrous atmosphere. About the same time our group has introduced alkyl halide alkylation of a chiral nickel (II) complex of a Schiff base of *(S)-O-*[(*N*-benzylprolyl)amino]benzophenone and glycine as a route to 6-l-[^18^F]FDOPA that proceed in the presence of potassium *tert*-butyrate at 80 °C and was amendable to automation [44]. However, the truly substantial development in the asymmetric synthesis has been achieved following the introduction of phase-transfer catalytic (PTC) reactions by Lemaire and co-workers [45] and Krasikova and co-workers [46]. Using various achiral substrate-PTC pairs for the key alkylation step, this approach was found to be suitable for generating 6-l-[^18^F]FDOPA in moderate to high radiochemical yields, enantiomeric purity of 98% and A_m_ as high as 0.75 TBq/µmol [47]. The details of this methodology can be found in the original publications [45,46,47,48] and summarized in the recent reviews of the synthesis of 6-l-[^18^F]FDOPA [33,35]. In general, the PTC-based multi-step approaches have also suffered from the problem of being difficult to implement in commercially available synthetic modules existing. However, following a decades worth of optimization [47,48] and, in a significant part, due to the progress in the automation technologies, a synthesis utilizing a chiral PTC—*O*-allyl-*N*-9-anthracenyl methyl-cinchonidinium bromide—and O’Donnel glycine substrate originating from the earlier work of Lemaire and co-workers [45,47,48] was fully automated on a commercial cassette-based synthesis module ‘AllInOne’ (Trasis) affording 6-l-[^18^F]FDOPA reproducibly and with an average RCY of >35% (decay uncorrected) with synthesis time of ca. 70 min, providing access to the Curie-level amounts of the radiotracer [49]. The successful automatization of this highly-complex 5-step synthesis was really a great achievement for PET radiochemistry.

**Scheme 3:**
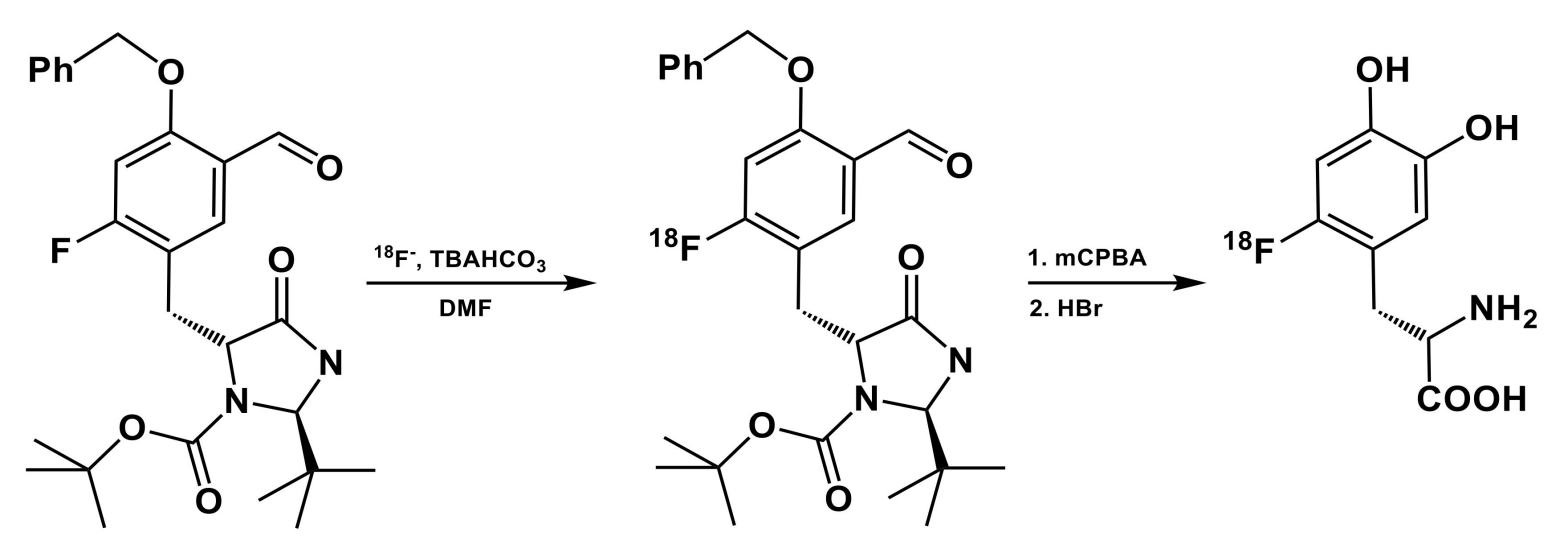
Radiosynthesis of 6-l-[^18^F]FDOPA by Baeyer–Villiger oxidation [50]; (1) mCPBA, CHCl_3_, 60 °C, 20 min; (2) HBr (47%), 150 °C, 30 min.

Recent attempts to implement this synthesis on an Ecker&Ziegler Modular-Lab Standard module were much less successful [33] than the original studies [47,48] or one employing a cassette-based platform [49]. Consequently, the authors [33] adopted another strategy, one based on a 3-step isotope-exchange synthesis developed by Ermert and co-workers [50] based on the Baeyer–Villiger oxidation with meta-chloroperbenzoic acid (Scheme 3). In the following study, the nitro-precursor for no-carrier added preparation of 6-l-[^18^F]FDOPA was introduced and patented [51]. According to the comparative study of the two methods [36], the 3-step synthetic approach using nitro-precursor (ABX 1336) was easier to automate, n.c.a. 6-l-[^18^F]FDOPA was obtained in average synthesis time of 114 min with RCY of 20 ± 1% and with molar activity of up to 2.2 GBq/μmol.

Still, considering the constraints imposed by the half-life of fluorine-18, methods for the introduction of the radionuclide into the desired molecular structure at the latest possible stage in the process to avoid radioactivity loss and radiation exposure to the personnel are preferable. Therefore, recent research in the field has been focused on the late-stage radiofluorination of non-activated (electron-rich) aromatic structures. In the past few years, several innovative ^18^F-labeling approaches have been introduced using, for example, iodonium salts, spirocyclic hypervalent iodine (III) complexes, organoborons and stannanes (for the reviews see [37,52,53,54,55,56,57]).

Their application in a development of simpler methods for the preparation of 6-l-[^18^F]FDOPA is discussed in the following chapters.

## 3. Late Stage Fluorinations

### 3.1. Iodonium Salts

An effective route to the incorporation of [^18^F]fluoride into electron-rich arenes is the use of diaryliodonium salts precursors, as pioneered by the Pike group [58]. Owing to their electron-deficient nature at the iodine center and simultaneous presence of an excellent leaving-group in the form of a Ph-I fragment, these compounds display exceptional reactivity when it comes to nucleophilic fluorination reactions. In the last decade both symmetric and non-symmetric diaryliodonium salt precursors have been evaluated for their usability for the introduction of a fluorine-18 label into a number of non-activated (hetero)aromatic substrates [59]. Good results have been achieved using diaryliodonium salts as precursors for the labeling of simple molecules, but aromatic nucleophilic substitution in structurally complex substrates remains a challenge, principally due to non-trivial synthesis of the respective precursors. In addition, precursor stability issues arise when trying to implement this synthetic methodology into routine productions for clinical applications.

**Scheme 4:**
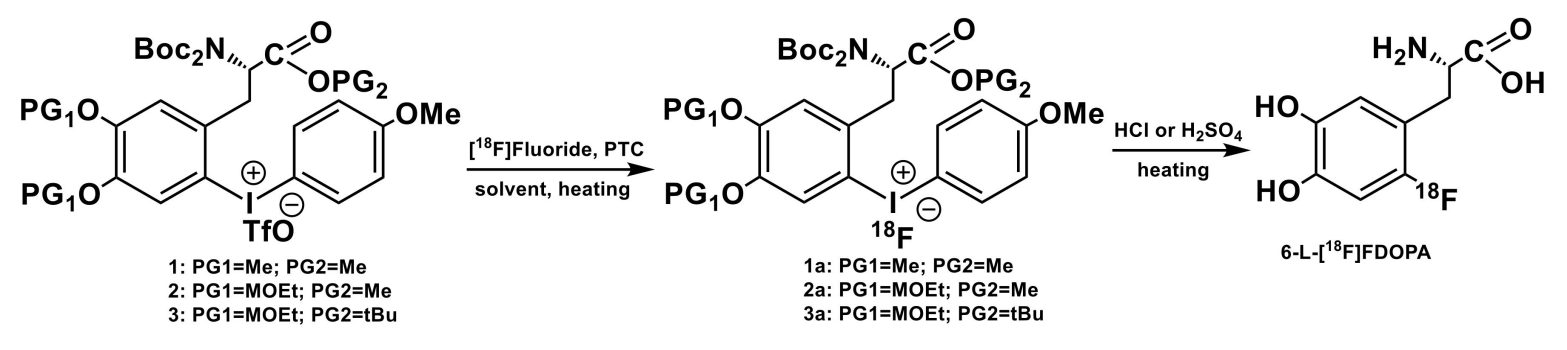
Preparation of 6-l-[^18^F]FDOPA via anisyl(aryl)iodonium salts precursors; (1) radiofluorination: [^18^F]TBAF, **1**, toluene, 130 °C, 20 min; deprotection: 48% HBr, 140 °C, 8 min [60]; (2) radiofluorination: [^18^F]fluoride, K_222_/K_2_CO_3_, **2**, diglyme, 140 °C, 5 min; deprotection: 3 M H_2_SO_4_, 140 °C, 5 min [63]; (3) radiofluorination, **3**, toluene, 105-110 °C, 10 min; deprotection: conc. HCl, 120 °C, 7 min [67].

In the last decade or so, there have been considerable efforts directed towards preparation 6-l-[^18^F]FDOPA via ^18^F-fluorination of a protected diaryliodonium salt precursors followed by acid deprotection [60,61,62,63,64,65,66,67]. DiMagno and co-workers [60,61] introduced anisyl(aryl)iodonium triflate salt as a suitable precursor for 6-l-[^18^F]FDOPA synthesis via TBA-mediated radiofluorination in toluene (Scheme 4, **1**). Consequently, the commercially-available analogue with ethoxymethyl (EOM) protecting groups on the catechol moiety, the ((*S*)-methyl-3-(4,5-bis(ethoxymethoxy)2-iodophenyl)-2-(di-(tert-butoxycarbonyl))amino)propanoate)(4-methoxyphenyl)-λ3-iodane trifluoromethanesulfonate (ALPDOPA) (Scheme 4, **2**) became available from Ground Fluor Pharmaceuticals [62]. According to the commercial report [62], the ^18^F-fluorination of ALPDOPA afforded 6-l-[^18^F]FDOPA in decay-corrected RCY of ca. 35% with synthesis time of 45 min; however, the synthesis details were not disclosed. Using the commercially-available ALPDOPA precursor, Elsinga and co-workers [63] were able to prepare 6-l-[^18^F]FDOPA in quality suitable for clinical applications with average RCY of 14% ± 4% (decay-corrected) in average molar activity of 35 ± 4 GBq/µmmol. Despite the simplicity of this two-steps one-pot synthesis method (kryptofix-mediated radiofluorination using 12 mg of ALDOPA precursor in dyglime at 140 °C for 5 min, followed by hydrolysis/deprotection with 3 M H_2_SO_4_ at 140 °C for 5 min), the synthesis time remained relatively long (117 ± 4 min) due to the need for two intermediate cartridge-based purifications. The n.c.a. radiotracer obtained was evaluated in an in-vivo model of xenografted neuroendocrine tumors demonstrating equal imaging quality compared to 6-l-[^18^F]FDOPA prepared by classic electrophilic radiofluorination of a stannyl precursor.

More recently, van Dam and co-workers have reported an automated version of this synthesis employing the ELIXYS FLEX/CHEM cassette-based synthesizer [64] affording 6-l-[^18^F]FDOPA in RCY of 4.5% ± 1.3% (decay-corrected) with an average synthesis time of 70 min. In addition to macroscale methods, the synthesis can also be conducted in a microdroplet reactor using only 0.11 mg of ALPDOPA precursor [65]. The radiotracer with high molar activity and RCY of 4.7 ± 1% was prepared on average within 30 min. Up to 15 MBq were available from a single synthesis, the amount was sufficient for the imaging of multiple mice.

Following the DiMagno approach, Wirth et al. suggested a simple synthetic scheme for the preparation of bench-stable anisyl(aryl)iodonium salts with bromine or iodine as the counter-ions for potential application in the synthesis of 6-l-[^18^F]FDOPA [66]. However, poor incorporation rates of [^18^F]fluoride into the precursor (2–5%) hampered their further investigation. The more recent experimental work by Maisonial-Besset et al. [67] on the ^18^F-labeling of 6-l-[^18^F]FDOPA employed anisyl(aryl)iodonium triflate precursor with easier-to-remove *tert*-butyl protecting group (Scheme 4, **3**). In that study, the authors followed the “minimalist” approach introduced by the Neumaier group [68] based on the direct elution of [^18^F]F^−^ from the anion-exchange cartridge using a solution of iodonium salt in methanol. The low-boiling MeOH was then evaporated; toluene was added to the residue and the reaction mixture was heated, furnishing the protected amino acid. Following acidic hydrolysis and semi-preparative HPLC purification, 6-l-[^18^F]FDOPA was obtained in the RCY of 27–38% (decay-corrected) with a synthesis time of 64 min. The synthesis was fully automated on a Raytest SynChrom R&D EVOIII synthesis module.

**Scheme 5:**
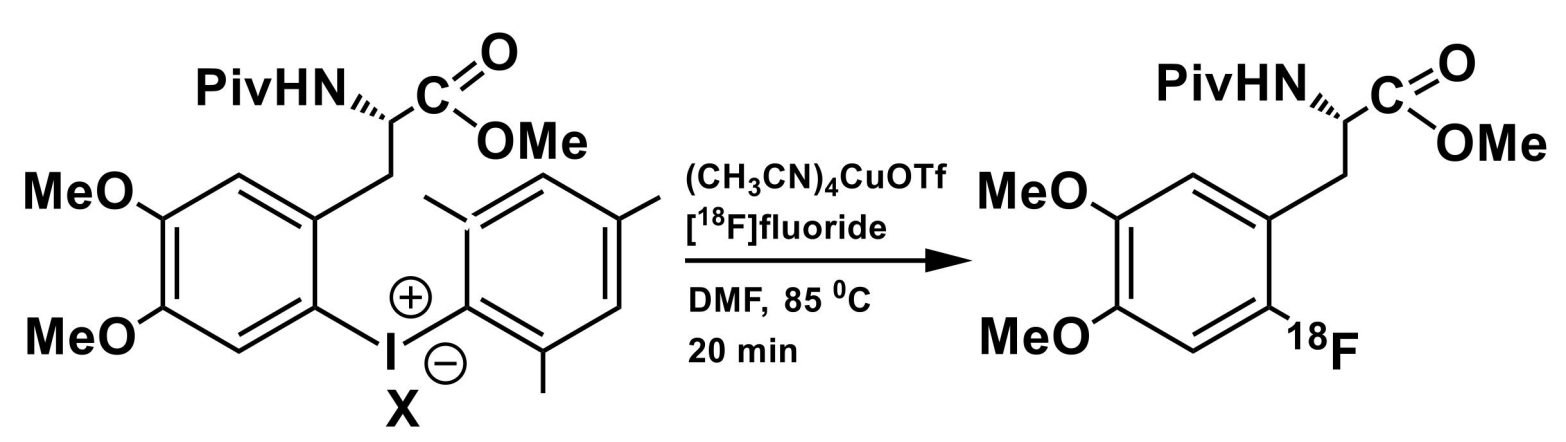
Preparation of protected 6-l-[^18^F]FDOPA via mesytil(aryl)iodonium salt precursor (X = Tos, BF_4_); conditions: 18-crown-6/K_2_CO_3_, azeotropic drying; [Mes-IDOPA]BF_4_ (12 μmol), (CH_3_CN)_4_CuOTf (12 μmol), DMF (0.7 mL) [70].

A significant step forward has been made by the Sanford group and Scott group [69,70] by designing a copper-mediated fluorination of (mesityl)(aryl)iodonium (MAI) salts (mesityl = mes = 2,4,6-trimethylphenyl) using commercially-available (CH_3_CN)_4_CuOTf complex and [^18^F]KF/18-crown-6 for the activation of the fluorine-18. Using a bulky mesityl group as an auxiliary forced the nucleophilic substitution towards less sterically hindered site on the arene ring [70]. Conducting radiofluorination in DMF or DMSO (instead of toluene [60,61,62], which is not well compatible with tubing materials in the module) in relatively mild reaction conditions are significant advantages for the implementation in modern automated synthesizers. This methodology was applied for the synthesis of several ^18^F-labeled aromatic amino acids including 6-l-[^18^F]FDOPA (Scheme 5) [70]. Radiofluorination of the respective MAI salt precursor affords protected amino acid with radiochemical conversion (RCC) of 17 ± 6% when using a tosylate counter ion and up to 30 ± 3% when a tetrafluoroborate is employed in the same role. This synthesis was fully automated on a GE TRACERlab FX FN automation platform; however, the protected 6-l-[^18^F]FDOPA from tetrafluoborate salt as the starting material was isolated with RCY (not-decay corrected) of only 1.1%. In this case, the deprotection step leading to the actual 6-l-[^18^F]FDOPA was not reported.

**Scheme 6:**
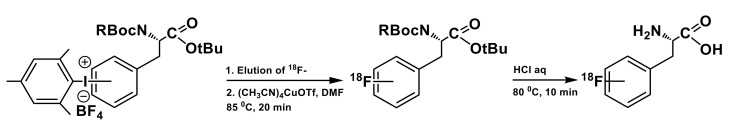
Preparation of 2, 3 and 4-[^18^F]labeled phenylalanines via minimalist protocol; radiofluorination: elution of [^18^F]fluoride from the ion exchange cartridge with a solution of the MAI precursor (21 µmol) in 20% MeOH in DMF (0.6 mL); Cu(MeCN)_4_OTf (21 µmol) in DMF (0.6 mL), 85 °C, 20 min; hydrolysis: 12 M HCl (0.5 mL), 80 °C, 10 min [71].

In follow-up studies by our group and Neumaier’s group [71], the minimalist protocol [68] was used for direct preparation of a series of 2,3- and 4-[^18^F]labeled phenylalanines in high yields, starting from respective MAI precursors (Scheme 6) without the use of PTC, base or any other additives. The [^18^F]fluoride retained on the anion-exchange resin was eluted with a solution of the MAI precursor in MeOH/DMF, followed by fluorination reaction in this combination of solvents. Use of alcohol as co-solvent for Cu-mediated ^18^F-fluorination of iodonium salts allowed for a substantial increase of radiofluorination efficiency leading to radiochemical conversion rates in the order of 60–86%, depending on position of fluorine in the aromatic ring. Notably, this iteration of minimalist protocol offers substantial practical benefits through the elimination of the azeotropic drying or any other solvent removal/replacement steps, thus significantly simplifying automation. Although this approach was proven to be efficient on the example of labeling a series of phenylalanines, it could potentially be used for preparation of 6-l-[^18^F]FDOPA as well. The synthesis of the respective MAI precursor has already been described [70], however, purification procedures required to produce it in sufficient amounts and desired quality are quite challenging. We have attempted synthesis of MAI tosylate salt with Boc-protection on the catechol moiety but failed to produce the precursor compound with high purity; consequently, a poor conversion of precursor into the ^18^F-labeleld product (<1%) was observed.

In general, the hypervalent I(III) reagents described above are not commercially available (with the exception of rather expensive ALPDOPA precursor) and must be prepared on site through multistep synthesis involving complex purification steps; the structural complexity and presence of impurities often leads to limited shelf life of precursors, which precludes their implementation in routine synthesis of 6-l-[^18^F]FDOPA for clinical applications. In addition, another important development that has shifted the focus of the radiochemistry research away from hypervalent iodine has been the introduction of a more practical method for the late-stage incorporation of fluorine-18 into non-activated arenes based on the easier-to-prepare, bench-stable arylboronic acid pinacol esters (ArylBPin) used in conjunction with Cu(OTf)_2_(py)_4_ catalyst [72].

### 3.2. Arylboronic Acid Pinacol Esters (ArylBPin)

**Scheme 7:**
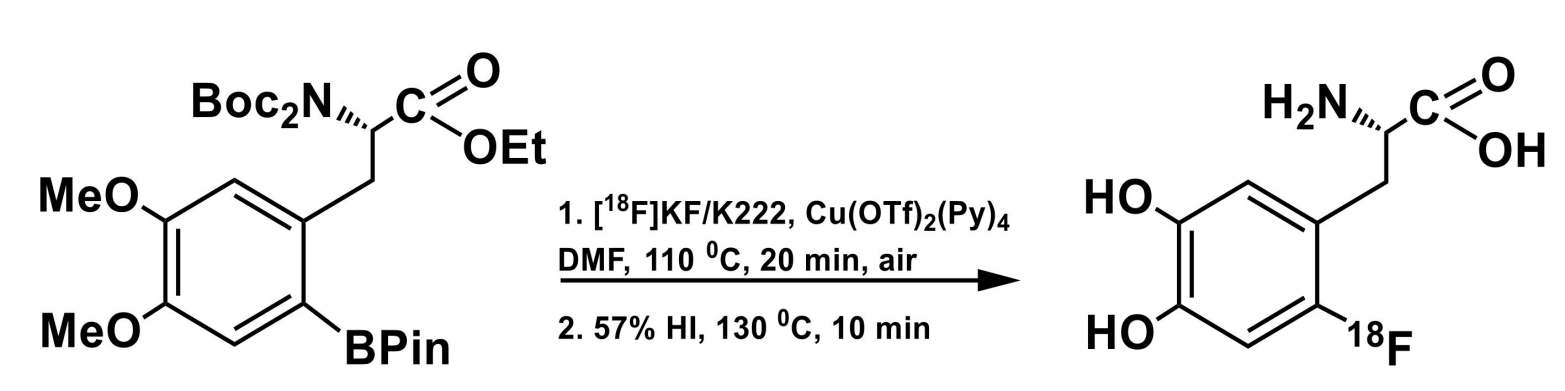
Synthesis of 6-l-[^18^F]FDOPA via Cu-mediated radiofluorination of an aryl pinacol-derived boronic ester (ArylBPin) precursor [72].

Among the late stage ^18^F-fluorinaton approaches, a copper-mediated labeling of aryl pinacol-derived boronic acid esters (ArylBPin) originally introduced by the Gouverneur group [72] offers a selective and widely applicable methodology for the introduction of [^18^F]fluoride into a large number of functionalized arenes. Inspired by the work of Sanford and co-workers on the copper-mediated fluorination of aryl trifluoroborates [73], Gouverneur et al. [72] adapted copper-assisted Chan-Lam type C-F cross-coupling reaction [74] to radiofluorinations of ArylBPin substrates in the presence of Cu(OTf)_2_(Py)_4_ complex as copper source. The scope of precursors to which the methodology is applicable has been further expanded to boronic acids [75], arylstannanes [76] and BPin-substituted Ni-based complexes [77,78]. However, the original copper-promoted radiofluorination of ArylBPin substrates [72] is, to date, the most versatile method accounting for the bulk of published labelings utilizing Cu-catalyzed fluorination (for the recent reviews, see [37,54,56]). The synthesis of 6-l-[^18^F]FDOPA via respective ArylBPin precursor [72] consisted of two steps (Scheme 7), with access to the atmospheric oxygen typically required for the fluorination reaction. Reactive species of [^18^F]fluoride were obtained eluting the radionuclide from an anion-exchange cartridge with a basic solution of K_2_CO_3_ and Kryptofix_2.2.2._ (3 mg of K_2_CO_3_ and 15 mg of K_2.2.2._) in a mixture of acetonitrile and water, followed by azeotropic drying to remove the water. In the original study [72], the [^18^F]KF/K_2.2.2._ was prepared on a Nanotech microfluidic system while the rest of the synthesis was performed on a semi-automated module with manual interventions.

The radiofluorination reaction was carried out using a 3:1 ratio of ArylBPin precursor to Cu-catalyst (60 and 20 µmol, respectively) affording protected amino acid in RCC of 55 ± 23% (n = 2, radioTLC). Aiming at full automation, the process was adapted to a conventional (not a microfluidic) module starting with 13 GBq of [^18^F]fluoride. As a result, total activity yield of 609 MBq of the formulated radiotracer was obtained that corresponded to a 12% RCY (decay corrected). The total synthesis time was not quoted, the procedure described contained several intermediate purification steps and required optimization. Automation of this method for producing large-scale batches of radiotracer has been the focus of significant efforts by several PET radiochemistry teams.

In a follow-up multi-center study directed by the Gouverneur’s group, the implementation of Cu-mediated synthesis of eight clinically-relevant radiotracers including 6-l-[^18^F]FDOPA on different automated platforms has been reported [79]. Considering the sensitivity of the Cu-mediated radiofluorination process to basic conditions reported by Neumaier and co-workers [80], the [^18^F]fluoride elution process was modified by replacing originally used K_2.2.2_/K_2_CO_3_ [72] with a less basic K_2.2.2._/K_2_C_2_O_4_/K_2_CO_3_ combination containing very small amounts of K_2_CO_3_ (16.7/5.4/0.72 µmol). Azeotropically-dried [^18^F]fluoride was allowed to react with an equimolar amounts of ArylBPin substrate and Cu(OTf)_2_(Py)_4_ (20 µmol each) at 120 °C for 20 min in DMF, followed by hydrolysis/deprotection reaction (57% HI, 150 °C, 10 min). The synthesis conducted on a Synthra automated module was not fully optimized, which explains the long synthesis time of 146 min and modest RCY of 8.7% (not decay corrected) [79].

With this new strategy for the introduction of fluorine-18 into non-activated arenes published, several studies have been undertaken to apply it to routine clinical production of radiotracers. Key optimization parameters investigated included the development of more effective protocols for preparing reactive [^18^F]fluoride using less-basic or neutral phase-transfer catalysts, optimization of reaction solvents and conditions, amount of labeling precursor used and ratios between the precursor and copper catalyst.

As was already mentioned, attempts to implement Cu-mediated fluorination for full-batch tracer production exposed the problem of copper catalyst stability in basic conditions. The amount of base used in the K_2.2.2._/K_2_CO_3_ approach can be minimized using the so-called “back-flushing protocol” where ^18^F^−^ is loaded onto the ion-exchange cartridge from the male side instead of the female side and eluted in the opposite direction. Using this protocol, introduced by the Neumaier’s group [80], high elution efficiency was achieved using only 0.43 μmol of K_2_CO_3_ instead of usual 20–25 μmol, thus enabling high-yield fluorination of several model ArylBPIn substrates. Combination of this protocol with [^18^F]fluoride elution using solution of tetraethylammonium bicarbonate (Et_4_NHCO_3_) in alcohol, suggested by the same group [81], has led to high recovery rates of the radionuclide from the ion-exchange cartridge and good fluorination efficiency of boronic ester precursors. Furthermore, the use of organic solutions of PTCs in the elution process allowed to simplify automation and shorten synthesis time by omitting azeotropic drying step. In combination with this back-flushing protocol, organic solutions of other PTCs such as tetrabutyl ammonium triflate (Bu_4_NOTf) [82] and pyridinium sulphonates [83] have been found to be effective for the preparation of reactive [^18^F]fluoride species for use in subsequent Cu-mediated fluorinations.

Base sensitivity of the Cu-complex and ArylBPin substrates notwithstanding, choice of the reaction solvent has been shown to have a significant impact on the outcome of this complex catalytic process. Cu-mediated radiofluorination of ArylBPin substrates most commonly carried out in either DMF or DMA [72]. More recently addition of alcohols as co-solvents has been found to be beneficial, leading to substantial increase in radiolabeling yields [81]. In the reverse sorption/elution protocol suggested in that study, ^18^F^–^ is eluted with a solution of Et_4_NHCO_3_ in alcohol (usually *n*-BuOH) into the solution of the appropriate precursor and Cu(OTf)_2_(Py)_4_ in DMA, followed by immediate heating of the resulting mixture affording the ^18^F-labeled product. This simple procedure eliminating azeotropic drying and solvents evaporation steps is advantageous for automation and allows to substantially shorten synthesis time. However, implementation of this approach for the synthesis of 6-l-[^18^F]FDOPA required high amount of the reactants (60 µmol of precursor and 53 µmol of Cu-catalyst) [81]. In a non-optimized manual example of the synthesis, 6-l-[^18^F]FDOPA was produced in RCY of 40 ± 4% (decay corrected). It should be noted that due to high amount of precursor the synthesis procedure involved intermediate cartridge-based purification steps and treatment of the ^18^F-labeled protected product with aqueous KHF_2_ solution (60 μmol)—something that is associated with difficulties in automation. Recently, [82] our group has reported preparation of 6-l-[^18^F]FDOPA via “alcohol-enhanced” fluorination of commercially-available (ABX, Germany) precursor-3,4-OMOM-6-(BPin)DOPA(Boc2)-OtBu possessing methoxymethyl (MOM) protecting groups on the catechol moiety. Using alcohol solution of Bu_4_NOTf (12.5 µmol in 600 µL of *i*-PrOH) employing back-flushing protocol lead to both high recovery rates of ^18^F-fluoride from the cartridge and high RCC (83 ± 6%, n = 7) for the following radiofluorination (5 µmol of Cu (OTf)_2_Py_4_, 8 µmol of ArylBPin, *i*-PrOH/DMA, 110 °C, 15 min, under air). In a semi-automated module with manual interventions 6-l-[^18^F]FDOPA was obtained with RCY of 20% (decay corrected, non-optimized) with average synthesis time of 80 min.

Using the same commercially available precursor the Scott’s group [84,85] has performed the first GMP-compliant synthesis of 6-l-[^18^F]FDOPA on a GE TRACERlab FX_FN_ automated platform. In general, the automated synthesis procedure was quite lengthy (110 min) and relatively complex due to the inclusion of the azeotropic drying step in the preparation of reactive [^18^F]fluoride, employing aqueous solution of Bu_4_NOTf and Cs_2_CO_3._ Compared with alcohol-enhanced conditions, radiofluorination in DMF proceeded with moderate RCC rate of 55%; also, the hydrolysis step required use of HCl in high concentration to ensure efficient deprotection. As a point of interest, in that study radiofluorination was carried out in the presence of Cu(OTf)_2_(Py)_4_ with the addition of 500 µmol of pyridine without provision for atmospheric oxygen usually associated with Cham-Lam coupling-like oxidation cycle [72]. This is an important finding as the standard automated modules for PET radiochemistry are flushed and operated with the inert gas and the introduction of the atmospheric oxygen can be problematic.

To summarize, although several radiolabeling protocols have been applied to Cu-mediated preparation of 6-l-[^18^F]FDOPA employing ArylBPin precursors, a full automation of the process still remains a challenging task. Transfer of methodology from manual or semi-automated modes to a fully automated process is often accompanied by a drop in isolated radiochemical yields, which are modest to begin with, a critical issue given high clinical demands for this radiotracer both in neurologic and oncologic applications. Among approaches investigated (Table 2) use of alcohol-enhanced conditions and neutral PTCs such as alkyl sulphonates for the preparation of the reactive [^18^F]F^−^ species appears to be one of the more promising avenues in view of application to fully automated production.

### 3.3. Organostannanes

**Scheme 8:**
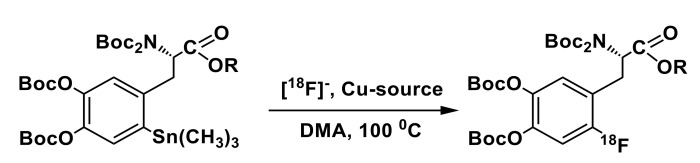
Cu-mediated synthesis of 6-l-[^18^F]FDOPA using aryl trimetyl stannane as labeling precursor. R = Me, ^18^F/KOTf/K_2_CO_3_; Cu(OTf)_2_, Py, 100 ^o^C, 30 min, DMA [76]; R = Et; ^18^F/Et_4_NOTf; Cu(OTf)_2_Py_4_; DMA; 100 °C, 10 min, air [86].

Similarly to Cu-mediated radiofluorination of organoboron compounds, in 2015 Sanford’s and Scott’s groups [76] have developed the first Cu-mediated nucleophilic radiofluorination of (hetero)aryl organostannanes employing [^18^F]KF along with catalyst generated in-situ from copper triflate and pyridine (Scheme 8). Arylstannanes are well-known in PET radiochemistry as precursors for direct ^18^F-labeling of non-activated arenes via electrophilic radiofluorination using [^18^F]F_2_ gas (Scheme 1). They are commercially available and have found validation as precursors for a number of ^18^F-fluorinated aromatic amino acids, including 6-l-[^18^F]FDOPA. A commercially available stannylated 6-l-[^18^F]FDOPA precursor with a single *t*-Boc protecting group on the amino function (TriBoc-l-DOPA methyl ester, 1340, ABX, Germany) was converted into di-Boc protected analogue in a one-step synthesis and radiolabeled in a reaction catalyzed by Cu(OTf)_2_ with additional pyridine. Reactive [^18^F]fluoride was obtained through elution of the radionuclide from the anion-exchange cartridge with a weakly basic combination of KOTf and K_2_CO_3_ (73/1 molar ratio). After fluorination reaction in DMA at 100 °C for 30 min, 6-l-[^18^F]FDOPA with protecting groups still in place was obtained in 56 ± 12% yield (manual operation, synthesis time not reported). These encouraging results [86] offer a potential route towards 6-l-[^18^F]FDOPA via nucleophilic substitution route, particularly given ease of access to the stannylated precursor.

Based on this proof-of-concept development [76], Neumaier’s group [86] undertook a systematic study of [^18^F]F^−^ recovery from target water for various routes of producing reactive fluoride species and radiolabeling process itself in order to determine optimal conditions of Cu-mediated fluorination of aryl stannanes for further implementation in fully automated synthesis. Of several PTC/base combinations investigated, use of Et_4_NOTf solution in methanol in conjunction with previously described back-flushing sorption-elution protocol was found to be the best. A fully protected ethyl ester precursor, *N,N,O,O’*-tetraBoc-6-(SnMe_3_)DOPA-OEt was prepared from the corresponding *N*-formyl precursor (6-Trimethylstannyl-l-DOPA, 1300, ABX, Germany) in >70% yield in a three step synthesis, and used for the following radiofluorination (Scheme 8). Using pre-formed Cu(OTf)_2_Py_4_ as a copper source radiofluorination in DMA afforded RCC of 54 ± 5% (n = 5) with a substrate to catalyst ratio of 1:2. Deprotection of the ^18^F-fluorinated ethyl ester precursor was achieved by heating with 48% HBr at 130 °C for 15 min affording the target amino acid in 37% RCY (decay corrected), with the synthesis time of ca. 60 min. For large-scale production (starting from 37–40 GBq of [^18^F]fluoride), the synthesis was fully automated using in-house designed double-reactor module. Interestingly, use of *n*-BuOH and other aliphatic alcohols as elution solvents and co-solvents to DMA in the fluorination step did not result in any reaction rate enhancement, as has been observed for Cu-mediated fluorinations of ArylBPin precursors [81].

Some issues may arise with the residual levels of the copper and tin in the injectable solution of the radiotracer. However, according to [86] the residual amounts of Cu and Sn in the final formulations of 6-l-[^18^F]FDOPA were well below the limits specified in the ICH guidelines (0.07–4.2 and 0.05–0.32 µg/batch vs. 340 and 640 µg/day, respectively [87]). For the determination of metal impurities, inductively coupled plasma-mass-spectrometry (ICP-MS) methods must be resorted to an outsource basis, as this kind of analytical equipment is not commonly available in clinical or even academic PET centers. As a viable alternative, Antuganov et al. [88] have recently proposed capillary electrophoresis (CE) as a simple, fast and cost-efficient analytical method allowing for concurrent determination of a number of potentially toxic impurities, including copper and tin, in buffered solutions of PET radiopharmaceuticals.

## 4. Summary and Conclusions

In recent years, numerous late-stage fluorination approaches for direct nucleophilic route of introduction of the fluoride-18 into non-activated aromatic substrates have been developed. These new methodologies have been evaluated for their potential to deliver amounts of 6-l-[^18^F]FDOPA suitable for clinical applications, as this tracer is highly sought after due to a wide application spectrum. While Cu-mediated radiofluorination of iodonium salts, arylpinacol boronates (ArylBPin) and arylstannanes discussed above displays significant potential for implementation in clinical productions, many of those approaches still require some, and in some cases, significant, improvement in terms of the activity yields and translation to full automation. Recently developed GMP compliant synthesis of 6-l-[^18^F]FDOPA via Cu-mediated fluorination of the commercially available MOM-protected ArylBPin precursor has been a major step forward; however, yield is still rather modest, especially given high demand for this radiotracer. In respect to Cu-mediated radiofluorination of mesytilaryl iodonium salts, the minimalist approach developed provided the simplest automation protocol for the preparation of 6-l-[^18^F]FDOPA. Nonetheless, challenges remain in the development of the synthesis procedure employing hypervalent I(III) precursors to put this method into clinical practice. Of the three late-stage fluorination approaches discussed above, Cu-mediated radiofluorination of trimethyl stannylated DOPA derivatives has shown the highest productivity, comparable to the best commercial cassette-based nucleophilic synthesis of 6-l-[^18^F]FDOPA (‘AllinOne’, Trasis) affording radiotracer reproducibly, with an average RCY of >35% (decay uncorrected). Radiofluorination of organostannanes in combination with azeotropic-drying free protocol using organic solutions of the phase-transfer catalyst on the ^18^F-recovery step is advantageous for automation. It is well-suited for the implementation on the modern flexible automated platforms such as GE TRACERlab FX_FN_ series, Synthra RN plus, Raytest SynChrom and others, which are now a common feature in numerous PET centers. Easy access to labeling substrates that can be simply prepared from commercially available stannylated precursors for 6-l-[^18^F]FDOPA is a further advantage, accelerating implementation of this method into routine GMP-compliant production.

## Figures and Tables

**Table 1:** Synthesis of 6-l-[^18^F]FDOPA via electrophilic radiofluorination of arylstannane precursor (R = CHO, Scheme 1).

^18^F source	Precursor, mg/µmol	Trapping/ Reaction Solvent	RCY, % (EOS); Synthesis Time, min	Molar Activity (A_m_)	Reference
^20^Ne(d,α)^18^F 0.35% F_2_	60/97	CFCl_3_	25 ± 3; 45 min	10.6 ± 2.3 GBq/mmol	[22]
^20^Ne(d,α)^18^F 0.25% F_2_	55–65/ 89–105	CHCl_3_	15 ± 5	8.5 ± 3.3 GBq/mmol	[24]
^18^O(p,n)^18^F 0.5% F_2_	30/48.5	CHCl_3_	23 ± 4	121 ± 27 GBq/mmol	[24]
^20^Ne(d,α)^18^F 0.5% F_2_	45/73	CDCl_3_	17.7 ± 2.3; 45 min	14.5 ± 3.5 GBq/mmol	[23]
^18^O(p,n)^18^F ^18^O-water target	6.2/10	acetone-d6	12.1 ± 3.7	3.4± 0.1 GBq/µmol	[31] *

RCY (EOS)—radiochemical yield at the end of synthesis (activity yield), not decay corrected; * [^18^F]F_2_ was prepared via post-target production; fluorinating agent—[^18^F]selectfluor bis(triflate).

**Table 2:** Synthesis of 6-l-[^18^F]FDOPA via fluorination of different ArylBPin precursors in the presence of Cu(OTf)_2_(py)_4_; fluorination at 110-120 °C, 15–20 min, under air.

Precursor Structure	[^18^F]Fluoride Elution	Precursor/ Cu-Complex, µmol	Reaction Solvent	Hydrolysis	RCY,% Synthesis Time	Ref.
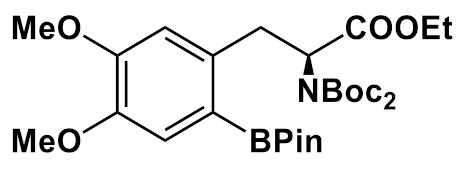	K_222_/K_2_C_2_O_4_/ K_2_CO_3_ /CH_3_CN/ H_2_O	20/20	DMF	57% HI, 150 °C, 10 min	8.7 146 min	[72]
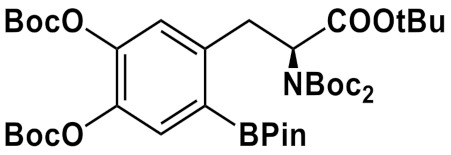	Et_4_NHCO_3_/ *n*-BuOH	60/53	DMA/ n-BuOH	12 M HCl,130 °C, 5 min	40 ± 4 ^a^ -	[81]
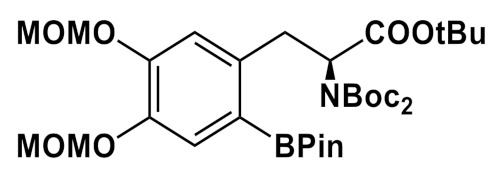	Bu_4_NOTf/ Cs_2_CO_3_/H_2_O	4/20 ^b^	DMF	12 M HCl, 0.25M ascorbic acid 130 °C, 5 min	6 ± 1 110 min	[84]
	Bu_4_NOTf/ *i*-PrOH	5/8	DMA/ *i*-PrOH	6 M HCl 120 °C, 10 min	12 80 min	[82]

RCY—radiochemical yield (activity yield), not decay corrected; ^a^ Radiochemical yield, decay corrected; ^b^ Addition of 500 µmol of pyridine; no air.
